# Effects of graded dietary levels of microalgae on growth performance, fatty acid composition, and meat quality in broilers

**DOI:** 10.1016/j.psj.2026.107629

**Published:** 2026-08-17

**Authors:** Jan Berend Lingens, Dana-Carina Schubert, Svenja Starke, Carsten Krischek, Christian Visscher, Amr Abd El-Wahab

**Affiliations:** a Institute for Animal Nutrition, University of Veterinary Medicine Hannover, Foundation, Bischofsholer Damm 15, D-30173 Hannover, Germany; b Nutrition and Clinical Nutrition Department, Faculty of Veterinary Medicine, Mansoura University, Mansoura 35516, Egypt; c Microganic GmbH, Melle, Germany; d Institute for Food Quality and Safety, University of Veterinary Medicine Hannover, Foundation, Bischofsholer Damm 15, D-30173 Hannover, Germany

**Keywords:** Microalgae, Broiler, Body weight, Fatty acids, Breast muscle quality

## Abstract

This study investigated the dose-dependent effects of two microalgae, *Chlorella vulgaris* and *Schizochytrium limacinum*, on broiler performance, health parameters, and product quality. A total of 280 mixed-sex Ross 308 broilers were randomly allotted to seven dietary treatments (five replicates, eight birds/replicate) for 35 days. A control group (CON) received a basal diet without algae. Three groups received the basal diet supplemented with *Chlorella vulgaris* at 0.5% (C0.5), 1.0% (C1.0), or 2.0% (C2.0), and three groups received *Schizochytrium limacinum* at 0.25% (S0.25), 0.5% (S0.5), or 1.0% (S1.0). Feed intake, body weight, foot pad health, intestinal morphology, and serum immunology were monitored. At day 36, birds were sampled for serum and liver fatty acid analysis and meat quality assessment. Final body weight and average daily gain were significantly higher (*p* < 0.05) in C0.5 (2526 g; 71.0 g/d) than in CON (2230 g; 62.5 g/d), a 13.3% improvement. *Schizochytrium limacinum* supplementation profoundly and dose-dependently altered fatty acid profiles: serum docosahexaenoic acid (DHA) was nearly nine-fold higher in S1.0 (161 µg/mL) than in CON (18.3 µg/mL), while arachidonic acid was lower (138 vs. 206 µg/mL). Liver DHA showed a similar pattern (6.21 vs. 0.80 g/kg dry matter). The S0.5 group showed enhanced humoral immune response to Newcastle Disease Virus vaccination (100% vs. 66.7% seropositivity in CON). Breast meat from C2.0 exhibited significantly higher yellowness (b*: 17.0 vs. 6.50 in CON), attributed to carotenoid deposition. Foot pad health and intestinal morphology were unaffected across treatments. In conclusion, low-dose *Chlorella vulgaris* (0.5%) improved growth performance, while *Schizochytrium limacinum* efficiently enriched tissues with omega-3 fatty acids in a dose-dependent manner. These findings demonstrate distinct and complementary applications of these microalgae as functional feed ingredients for targeted improvements in poultry production and product quality.

## Introduction

Integrating functional ingredients into poultry nutrition is a strategic priority, driven by both environmental concerns and market demand for animal products enriched with beneficial compounds - such as omega-3 fatty acids and natural pigments - through biologically sourced feed ingredients rather than synthetic additives. Microalgae represent a promising class of such ingredients, with a nutritional profile that can simultaneously address sustainability goals and bird health (Martins et al., 2021; Saadaoui et al., 2021). Their cultivation circumvents competition for arable land, and certain species are notably efficient at sequestering carbon dioxide (Kiseleva et al., 2024; Winckelmann et al., 2015). For the broiler industry, microalgae, such as *Chlorella,* used at a high level, did not impair birds' growth performance, having an added value on nutritional quality of meat (Alfaia et al., 2021).

The biological effects of dietary microalgae are intrinsically linked to their specific biochemical composition. The green alga *Chlorella* spp. is a recognized source of proteins, carotenoid pigments, and polysaccharides like β-glucans, which are associated with immunomodulatory and antioxidant activities (Kang et al., 2013; Ritu et al., 2023). In contrast, the heterotrophic marine protist *Schizochytrium* spp. is primarily valued for its exceptional capacity to synthesize and accumulate very long-chain omega-3 polyunsaturated fatty acids (PUFAs), particularly docosahexaenoic acid (DHA) (Park et al., 2015; Valente et al., 2021). This fundamental compositional difference implies that these two algae likely influence broiler physiology through distinct mechanisms and may therefore have different optimal application levels in feed. Briefly, *Chlorella* spp. and *Schizochytrium* spp. differ markedly in their nutritional composition, with *Chlorella* characterized by high protein and pigment contents (Saadaoui et al., 2021), whereas *Schizochytrium* is predominantly a lipid-rich microalga providing high concentrations of PUFAs, particularly DHA (Ao et al., 2015). Previous studies have shown that these compositional differences are associated with distinct physiological effects in poultry, including growth performance, immune modulation, oxidative status, and tissue fatty acid deposition (An et al., 2016; Kang et al., 2013; Yan and Kim, 2013). Consequently, these algae are expected to influence broiler physiology via different nutritional and metabolic pathways, which may necessitate species-specific optimal inclusion levels in poultry diets.

While numerous studies have reported on the inclusion of single algal species in broiler diets, results regarding growth performance, health, and product quality are often inconsistent (Long et al., 2018; Pestana et al., 2020). Consequently, it remains difficult for producers to determine economically viable inclusion levels that maximize specific benefits, such as growth, product quality, or health support, without negatively affecting feed efficiency. A major source of this variability is the lack of systematic dose-response data. Against this background, establishing a clear relationship between algal inclusion level and physiological response represents a key objective of the present study, as it is a prerequisite for developing evidence-based feeding recommendations. Without such data, identifying inclusion levels that maximize desired outcomes while maintaining feed efficiency and economic feasibility remains challenging.

Preliminary research comparing various algal species at a standard inclusion rate identified *Chlorella vulgaris* (with a mechanically disrupted cell wall) and *Schizochytrium limacinum* as particularly influential on meat color and tissue fatty acid profiles, respectively (Abd El-Wahab et al., 2025). This fact indicated their high potential for targeted applications, but left the optimal inclusion level for each effect undefined. Building directly upon these findings, the present study was designed to characterize the dose-response relationships for these two selected microalgae. The objective was to evaluate the effects of graded dietary levels of *Chlorella vulgaris* and *Schizochytrium limacinum* on broiler growth performance, fatty acid contents (as reflected in serum and liver profiles), and meat quality attributes. This work aims to provide foundational data necessary for the targeted and efficient use of these specific microalgae in broiler nutrition.

## Materials and methods

### Ethical approval

The animal trial was conducted in compliance with German animal welfare regulations. As the study involved no interventions prior to the scheduled dissection for sample collection, formal approval under the German Animal Welfare Act (Tierschutzgesetz - TierSchG) was not required (§ 7, paragraph 2, sentence 3). The slaughter procedure for scientific purposes was approved by the Animal Welfare Officer of the University of Veterinary Medicine Hannover, Foundation, Hannover, Germany (reference: TiHo-T-2023-1).

### Animals, housing, and experimental design

A total of 280 one-day-old Ross 308 broiler chickens of both sexs (BWE-Brüterei Weser-Ems GmbH & Co. KG, Visbeck-Richterfeld, Germany) were used in a 35-day feeding trial, with sampling performed at the end of the experimental period (i.e., at the beginning of day 36). Upon arrival, chicks were randomly allocated to seven dietary treatment groups. Each group consisted of five replicate pens, with eight birds per pen (*n*=40 birds per treatment, *N*=280 total).

Birds were housed in floor pens bedded with wood shavings, in accordance with applicable European Union animal welfare regulations. In each pen, a diagonal nipple drinking line (two nipples per pen) provided *ad libitum* access to water, and a single hanging feeder provided *ad libitum* access to feed (Klaus Gritsteinwerk GmbH & Co. KG, Bünde, Germany). Additionally, an infrared heat lamp was installed in each pen. Room ventilation was achieved via a vacuum system. A lighting schedule of 16L:8D was maintained, with continuous light provided for the first 72 hours.

### Dietary treatments

The experiment was designed as a dose-response study with two microalgae species selected based on a prior screening experiment (Abd El-Wahab et al., 2025). The seven dietary treatments consisted of a control group fed a basal diet without microalgae supplementation (CON), and six experimental groups. Three groups received the basal diet supplemented with *Chlorella vulgaris* (cell wall mechanically disrupted by the manufacturer to enhance bioaccessibility; Microganic GmbH, Melle, Germany) at inclusion levels of 0.5% (C0.5), 1.0% (C1.0), and 2.0% (C2.0). The remaining three groups received the basal diet supplemented with *Schizochytrium limacinum* (Microganic GmbH, Melle, Germany) at inclusion levels of 0.25% (S0.25), 0.5% (S0.5), and 1.0% (S1.0). *Chlorella* exhibits a proximate composition of 55.6% crude protein, 10.8% crude fat, 9.7% ash, and 1.1% crude fiber, whereas *Schizochytrium* comprises 25.6% crude protein, 37.8% crude fat, 8.55% ash, and 0.7% crude fiber.

For performance evaluation, feed and water intake were recorded at the same intervals as body-weight measurements (days 0, 7, 11, 14, 21, 28, and 35). Accordingly, phase-specific performance parameters were calculated for the starter period (days 0-11), grower period (days 11-21), and finisher period (days 21-35). For each period, feed and water intake were summed across the corresponding measurement intervals and expressed per bird per day. Average daily gain (ADG) was calculated from body-weight gain over the respective period. Feed conversion ratio (FCR) was calculated as feed intake divided by body-weight gain, and the water-to-feed ratio was calculated as water intake divided by feed intake.

The basal mash diets, formulated based on wheat, soybean meal, and corn, were produced by a commercial feed mill (BEST 3 Geflügelernährung, Twistringen, Germany). Microalgae powders were thoroughly blended into the respective basal mash at the Institute for Animal Nutrition before pelleting. The analyzed chemical composition of the unsupplemented basal diets is presented in Table 1.

**Table 1:** Analyzed chemical composition of of the unsupplemented basal diets (g per kg dry matter).

Parameter/Dietary phase	Starter	Grower	Finisher
Dry matter	881	886	886
Crude ash	50.5	57.2	38.3
Crude fiber	33.4	31.9	32.3
Crude fat	53.7	65.7	82.0
Crude protein	233	241	201
Calcium	8.35	9.56	4.86
Phosphorus	5.52	6.16	4.32
Starch	493	456	507
Sugar	48.4	48.9	46.6
Essential amino acids			
Arginine	15.2	16.5	12.9
Glycine	10.1	10.2	8.48
Histidine	5.69	5.83	4.85
Isoleucine	9.65	10.1	8.24
Leucine	17.6	17.9	14.7
Lysine	13.6	15.5	11.6
Methionine	5.38	7.13	4.51
Phenylalanine	11.2	11.3	9.28
Threonine	7.98	9.09	8.14
Valine	11.1	11.9	10.0
Non-essential amino acids			
Alanine	10.2	10.6	8.62
Cysteine	4.15	4.30	3.41
Proline	15.7	15.4	14.1
Serine	11.3	11.3	9.48
Tyrosine	7.58	7.69	6.23
Aspargine	21,3	21,8	16,8
Metabolizable energy (MJ/kg)	14.3	14.2	15.0

### Analyses, data, and sample collection

*Chemical analysis of feed*. Feed analysis was conducted following established protocols of the Association of German Agricultural Analytic and Research Institutes (Naumann and Bassler, 2012). The dry matter (DM) content was determined by oven-drying samples at 103°C to a constant weight. Ash content was measured by incineration in a muffle furnace at 600°C for six hours. Total nitrogen was assessed by using a rapid Max N exceed® analyzer (Elementar Analysensysteme GmbH, Langenselbold, Germany), and crude protein was calculated by multiplying the nitrogen value by 6.25. Crude fat was extracted and quantified by automated hydrolysis and solvent extraction (Hydrotherm and SOXTHERM 416 systems, C. Gerhardt GmbH & Co. KG, Königswinter, Germany). The crude fiber fraction was determined by sequential digestion in boiling dilute sulfuric acid and potassium hydroxide. The resulting residue was filtered, dried, and ashed, with the loss on ignition being measured. Starch concentration was determined by polarimetric analysis (Schmidt + Haensch GmbH & Co., Berlin, Germany), while sugar content was evaluated using the Luff-Schoorl method.

Amino acid composition was analyzed by ion-exchange chromatography (Biochrom 30+, Biochrom Ltd., Harvard Bioscience Inc., Cambridge, UK). Fatty acid composition was determined as fatty acid methyl esters (FAME) by gas chromatography. Samples were subjected to direct transesterification according to Lepage and Roy (1986), in which lipids were converted to methyl esters in a methanol–benzene mixture (4:1, v/v) with acetyl chloride in the presence of tridecanoic acid (C13:0) as an internal standard. The reaction was carried out at 100°C for 1 h. After cooling, the mixture was neutralized with 6% K₂CO₃ solution and centrifuged, and an aliquot of the upper benzene phase was collected for analysis. FAME were quantified by gas chromatography (Gaschromatograph Trace 1300, Thermo Fisher Scientific GmbH, Dreieich, Germany) with flame ionization detection (GC-FID).

*Performance*. Birds were weighed individually at days 0, 7, 11, 14, 21, 28, 35. Feed and water intakes were recorded concurrently to calculate average daily feed intake (ADFI), ADG, FCR, and water-to-feed ratio. Litter and fresh excreta DM were determined weekly from standardized sampling points (El-Wahab et al., 2020).

*Excreta, litter dry matter and foot pad health*. Foot pad health was assessed weekly for all birds using the lesion scoring system of Mayne et al. (2007), where 0 indicates no lesion and 7 represents severe necrosis (>50% of foot pad).

*Slaughter and sampling.* At day 36 (after completing feeding experimental period and at the beginning of day 36), 15 birds per treatment (three randomly selected from each of the five replicate pens) were humanely slaughtered. Birds were humanely killed in accordance with Annex IV of Directive 2010/63/EU on the protection of animals used for scientific purposes. Specifically, broilers were stunned via a percussive blow to the head (concussion), followed immediately by exsanguination. Blood was collected, and serum was separated by centrifugation (3000 × g, 10 min) and stored at -20°C for subsequent analysis. Tissue samples from the jejunum (mid-section) and ileum (oral to the ileo-cecal junction) were collected and preserved in 4% formaldehyde for histomorphometry. The left *Musculus pectoralis superficialis* (breast fillet) was excised for meat quality analysis. This analysis was performed on a subset of the dietary groups, specifically the CON and the treatment groups representing the lowest and highest inclusion levels for each algal species (C0.5, C2.0, S0.25, and S1.0), to evaluate potential dose-response effects; intermediate dose groups (C1.0 and S0.5) were not included in the meat quality assessment.

*Serum antibody response*. Serum antibody response. Broilers were vaccinated once via drinking water at day 14 of life with a live vaccine (Nobilis Ms5+Clone 30; Stamm Clone 30 for NDV). Serum antibody titers against Newcastle Disease Virus (NDV) was quantified using commercial indirect Enzyme-Linked Immunosorbent Assay (ELISA) kits (BioChek BV, Reeuwijk, The Netherlands).

*Intestinal histomorphometry*. Fixed intestinal samples were processed by standard paraffin embedding, sectioning (4 µm), and hematoxylin-eosin staining. Villus height (VH), crypt depth (CD), and villus width (VW) were measured using image analysis software on a Zeiss Axioscope microscope. The villus height to crypt depth ratio (VH:CD) was calculated.

*Serum and hepatic fatty acid composition*. Fatty acid composition was analyzed as fatty acid methyl esters using gas chromatography with flame ionization detection (GC-FID; Trace 1300, Thermo Fisher Scientific GmbH, Dreieich, Germany). Samples were subjected to direct transesterification using a methanol-benzene mixture (4:1, v/v) with acetyl chloride, and tridecanoic acid (C13:0) as an internal standard. The reaction was conducted at 100°C for 1 h. After cooling, samples were neutralized with 6% K₂CO₃, centrifuged, and the upper phase was collected for chromatographic analysis.

*Meat quality*. At the Institute for Food Quality and Food Safety, pH and electrical conductivity (EC) of breast muscle were measured 24 hours post mortem using a portable pH meter (Portamess® 911 pH, Knick GmbH, Berlin) and an LF-Star® conductivity meter (Matthaeus GmbH, Pöttmes, Germany), respectively. Instrumental color (CIE L, a, b*) was assessed on a fresh muscle surface using a Minolta CR-400 colorimeter (Konica Minolta GmbH, Langenhagen, Germany).

### Statistical analysis

Data were analyzed using SAS® Enterprise Guide® 7.1 (SAS Institute Inc., Cary, NC, USA). Performance data (ADFI, FCR) and litter DM were analyzed at pen level (*n*=5). Body weight (BW), histology, fatty acids, and meat quality data were analyzed at individual bird level (*n*=15). Sample sizes were determined based on previous similar studies and institutional guidelines, with 15 birds per treatment providing adequate statistical power for detecting meaningful biological differences. The effect of dietary treatment was evaluated using a one-way ANOVA. For normally distributed data (Shapiro-Wilk test), differences between means were separated using the Ryan-Einot-Gabriel-Welsch multiple range test. A probability level of *p* < 0.05 was considered statistically significant. Seropositivity rates for NDV were compared between groups using Fisher's exact test, appropriate for the small sample size and categorical nature of the data.

## Results

### Growth performance

Feed intake across the different dietary phases and overall periods is presented in Table 2. During the starter phase, feed intake differed significantly among groups (*p* < 0.05): the S0.5 group had the lowest intake (27.9 ± 0.89 g/d), significantly lower than CON (31.1), C0.5 (33.8), and C2.0 (30.7); C1.0 (30.1) was also significantly lower than C0.5. No significant differences were observed during the grower, finisher, or total periods (*p* > 0.05)

**Table 2:** Feed intake during the dietary phases as well as over the entire phases depending on the feeding group, given in g/animal/day (Mean ± SD).

Group^1^/Dietary phase	Starter	Grower	Finisher	Total
CON	31.1^a^^,^^b^ ± 2.18	79.5^a^ ± 7.02	137^a^ ± 9.02	87.1^a^ ± 5.28
C0.5	33.8^a^ ± 2.26	89.3^a^ ± 5.77	148^a^ ± 8.35	94.8^a^ ± 3.91
C1.0	30.1^b^ ± 0.52	80.6^a^ ± 2.35	132^a^ ± 5.89	85.4^a^ ± 2.25
C2.0	30.7^a^^,^^b^ ± 1.60	82.7^a^ ± 9.42	141^a^ ± 10.5	89.8^a^ ± 6.19
S0.25	30.6^a^^,^^b^^,c^ ± 1.85	85.7^a^ ± 5.23	140^a^ ± 10.7	90.2^a^ ± 5.77
S0.5	27.9^c^ ± 0.89	80.5^a^ ± 4.79	138^a^ ± 1.93	86.8^a^ ± 2.03
S1.0	30.3^a^^,^^b^^,c^ ± 1.45	86.6^a^ ± 4.14	140^a^ ± 13.8	90.2^a^ ± 6.09

a Different superscripts within column mark significant differences between the groups (*p* < 0.05).

b Different superscripts within column mark significant differences between the groups (*p* < 0.05).

1 CON, control (basal diet without microalgae); C0.5, C1.0, C2.0, basal diet supplemented with *Chlorella vulgaris* at 0.5%, 1.0%, and 2.0%, respectively; S0.25, S0.5, S1.0, basal diet supplemented with *Schizochytrium limacinum* at 0.25%, 0.5%, and 1.0%, respectively.

The water-to-feed intake ratio throughout the experimental phases is detailed in Table 3. Statistical analysis revealed no significant differences between any of the dietary treatment groups at any stage of the trial, including the overall period (*p* > 0.05).

**Table 3:** Water-to-feed intake ratio during the dietary phases as well as over the entire phases depending on the feeding group (Mean ± SD).

Group^1^/Dietary phase	Starter	Grower	Finisher	Total
CON	2.66 ± 0.26	2.25 ± 0.09	1.74 ± 0.11	1.97 ± 0.04
C0.5	2.65 ± 0.24	2.14 ± 0.17	1.85 ± 0.06	2.01 ± 0.09
C1.0	2.70 ± 0.12	2.25 ± 0.09	1.92 ± 0.16	2.09 ± 0.13
C2.0	2.69 ± 0.17	2.28 ± 0.32	1.83 ± 0.13	2.04 ± 0.13
S0.25	2.68 ± 0.22	2.06 ± 0.10	1.91 ± 0.18	2.03 ± 0.13
S0.5	2.79 ± 0.21	2.30 ± 0.26	1.81 ± 0.07	2.03 ± 0.08
S1.0	2.56 ± 0.25	1.97 ± 0.19	2.00 ± 0.13	2.05 ± 0.14

Statistical evaluation revealed no significant differences between the groups (*p* > 0.05).

1 CON, control (basal diet without microalgae); C0.5, C1.0, C2.0, basal diet supplemented with *Chlorella vulgaris* at 0.5%, 1.0%, and 2.0%, respectively; S0.25, S0.5, S1.0, basal diet supplemented with *Schizochytrium limacinum* at 0.25%, 0.5%, and 1.0%, respectively.

The body weight of broilers fed graded levels of *Chlorella vulgaris* or *Schizochytrium limacinum* is presented in Table 4. Over the 35-day experimental period, final BW was significantly influenced by dietary treatment (*p* < 0.05). Birds receiving the diet supplemented with 0.5% *Chlorella vulgaris* (C0.5) achieved a significantly higher final BW (2526 ± 296 g) and overall ADG (71.0 ± 2.59 g/d) compared to the CON (2230 ± 265 g; 62.5 ± 3.87 g/d). Although a numerically higher final BW was also observed for the C2.0, S0.25, and S1.0 groups, these did not differ statistically from the CON (Table 4). In contrast, the group receiving 0.5% *Schizochytrium limacinum* (S0.5) exhibited a transient reduction in BW during the grower phase, though this effect was no longer apparent by day 35. The ADG is presented in Table 5. During the finisher phase (days 28-35) and over the entire 35-day period, the ADG of the C0.5 group was significantly higher than that of the CON, C1.0, and S0.5 groups (*p* < 0.05). The C2.0, S0.25, and S1.0 groups showed numerically higher ADG than the CON but without statistical significance (Table 5). No significant differences in overall FCR (total period) were observed among the dietary treatments (*p* > 0.05) (Table 6).

**Table 4:** Body weight in g during the experimental period (Mean ± SD).

Group^1^	Day 0	Day 7	Day 11	Day 14	Day 21	Day 28	Day 35
CON	42.3^a^ ± 2.76	188^a^^,^^b^ ± 20.9	357^a^^,^^b^ ± 42.7	514^a^^,^^b^ ± 64.4	1020^a^^,^^b^ ± 126	1577^b^ ± 196	2230^b^ ± 265
C0.5	42.6^a^ ± 2.93	191^a^ ± 16.7	368^a^ ± 38.6	545^a^ ± 56.2	1115^a^ ± 122	1760^a^ ± 209	2526^a^ ± 296
C1.0	42.8^a^ ± 2.85	189^a^^,^^b^ ± 14.0	356^a^^,^^b^ ± 28.8	516^a^^,^^b^ ± 43.9	1006^a^^,^^b^ ± 101	1585^b^ ± 172	2257^b^ ± 259
C2.0	42.0^a^ ± 2.40	186^a^^,^^b^ ± 18.9	353^a^^,^^b^ ± 36.6	513^a^^,^^b^ ± 60.3	1026^a^^,^^b^ ± 122	1621^a^^,^^b^ ± 201	2335^a^^,^^b^ ± 298
S0.25	42.0^a^ ± 3.20	183^a^^,^^b^ ± 13.3	343^a^^,^^b^ ± 32.7	519^a^^,^^b^ ± 55.6	1027^a^^,^^b^ ± 200	1669^a^^,^^b^ ± 190	2381^a^^,^^b^ ± 296
S0.5	41.9^a^ ± 3.02	172^c^ ± 14.4	329^b^ ± 35.7	487^a^ ± 53.1	990^b^ ± 109	1557^b^ ± 208	2243^b^ ± 297
S1.0	41.6^a^ ± 3.54	177^b^^,c^ ± 14.9	333^b^ ± 31.1	493^a^ ± 53.8	997^a^^,^^b^ ± 115	1603^a^^,^^b^ ± 222	2328^a^^,^^b^ ± 328

a Different superscripts within column mark significant differences between the groups (*p* < 0.05).

b Different superscripts within column mark significant differences between the groups (*p* < 0.05).

1 CON, control (basal diet without microalgae); C0.5, C1.0, C2.0, basal diet supplemented with *Chlorella vulgaris* at 0.5%, 1.0%, and 2.0%, respectively; S0.25, S0.5, S1.0, basal diet supplemented with *Schizochytrium limacinum* at 0.25%, 0.5%, and 1.0%, respectively.

**Table 5:** Average daily gain during the experimental period depending on the feeding group, given in g/animal/day (Mean ± SD).

Group^1^	Starter	Grower	Finisher	Total
CON	28.6^a^^,^^b^ ± 2.36	66.3^a^ ± 6.07	86.4^c^ ± 4.08	62.5^b^ ± 3.87
C0.5	29.6^a^ ± 1.10	74.6^a^ ± 3.11	101^a^ ± 4.11	71.0^a^ ± 2.59
C1.0	28.4^a^^,^^b^ ± 0.53	65.0^a^ ± 1.76	89.4^b^^,^^c^ ± 4.18	63.3^b^ ± 5.16
C2.0	28.2^a^^,^^b^ ± 1.33	67.3^a^ ± 3.52	93.5^a^^,^^b^^,^^c^ ± 3.58	65.5^a^^,^^b^ ± 2.76
S0.25	27.4^a^^,^^b^ ± 1.06	68.4^a^ ± 9.18	96.7^a^^,^^b^ ± 3.73	66.8^a^^,^^b^ ± 3.50
S0.5	26.1^b^ ± 1.47	66.1^a^ ± 2.45	89.5^b^^,^^c^ ± 2.65	62.9^b^ ± 1.92
S1.0	26.5^b^ ± 1.22	66.4^a^ ± 4.68	95.1^a^^,^^b^^,^^c^ ± 8.29	65.3^a^^,^^b^ ± 4.96

a Different superscripts within column mark significant differences between the groups (*p* < 0.05).

b Different superscripts within column mark significant differences between the groups (*p* < 0.05).

c Different superscripts within column mark significant differences between the groups (*p* < 0.05).

1 CON, control (basal diet without microalgae); C0.5, C1.0, C2.0, basal diet supplemented with *Chlorella vulgaris* at 0.5%, 1.0%, and 2.0%, respectively; S0.25, S0.5, S1.0, basal diet supplemented with *Schizochytrium limacinum* at 0.25%, 0.5%, and 1.0%, respectively.

**Table 6:** Feed conversion ratio during the dietary phases as well as over the entire phases depending on the feeding group (Mean ± SD).

Group^1^/Dietary phase	Starter	Grower	Finisher	Total
CON	1.09 ± 0.10	1.20 ± 0.02	1.58 ± 0.14	1.39 ± 0.08
C0.5	1.12 ± 0.07	1.19 ± 0.24	1.46 ± 0.04	1.34 ± 0.02
C1.0	1.06 ± 0.03	1.24 ± 0.03	1.48 ± 0.02	1.35 ± 0.02
C2.0	1.09 ± 0.03	1.22 ± 0.09	1.51 ± 0.06	1.37 ± 0.04
S0.25	1.12 ± 0.07	1.27 ± 0.13	1.45 ± 0.08	1.35 ± 0.02
S0.5	1.07 ± 0.05	1.22 ± 0.07	1.54 ± 0.03	1.38 ± 0.02
S1.0	1.15 ± 0.09	1.31 ± 0.09	1.47 ± 0.05	1.38 ± 0.05

Statistical evaluation revealed no significant differences between the groups (*p* > 0.05).

1 CON, control (basal diet without microalgae); C0.5, C1.0, C2.0, basal diet supplemented with *Chlorella vulgaris* at 0.5%, 1.0%, and 2.0%, respectively; S0.25, S0.5, S1.0, basal diet supplemented with *Schizochytrium limacinum* at 0.25%, 0.5%, and 1.0%, respectively.

### Dry matter content of excreta/litter

The DM content of excreta and litter measured at weekly intervals is summarized in Table 7. Statistical evaluation revealed no significant differences between any of the treatment groups at any time point throughout the study (*p* > 0.05), indicating that microalgae supplementation did not affect these parameters.

**Table 7:** Excreta and litter dry matter contents (g/kg) during the experimental period (Mean ± SD).

Group^1^	Day 0	Day 7	Day 14	Day 21	Day 28	Day 35
Excreta						
CON	209 ± 1.0	205 ± 8.36	204 ± 16.2	225 ± 26.6	227 ± 13.4	209 ± 16.0
C0.5	208 ± 27.3	204 ± 9.69	204 ± 8.61	217 ± 13.0	248 ± 8.66	208 ± 27.3
C1.0	198 ± 14.3	208 ± 15.2	205 ± 7.49	230 ± 20.5	211 ± 46.1	198 ± 14.3
C2.0	209 ± 19.6	213 ± 11.7	213 ± 13.7	234 ± 23.1	209 ± 10.2	209 ± 19.6
S0.25	192 ± 11.0	212 ± 16.7	205 ± 9.90	214 ± 16.5	217 ± 11.3	192 ± 11.0
S0.5	215 ± 14.6	206 ± 7.82	205 ± 9.52	226 ± 19.4	218 ± 24.6	215 ± 14.6
S1.0	219 ± 13.7	216 ± 9.36	216 ± 10.3	198 ± 19.6	223 ± 18.8	219 ± 13.7
Litter						
CON	876 ± 45.3	828 ± 55,3	751 ± 29,8	800 ± 37.1	768 ± 7.58	876 ± 45.3
C0.5	864 ± 31.2	800 ± 30.7	755 ± 48.7	762 ± 34.9	747 ± 74.0	864 ± 31.2
C1.0	865 ± 37.0	824 ± 24.4	776 ± 27.6	773 ± 29.9	782 ± 33.7	865 ± 37.0
C2.0	850 ± 47.8	814 ± 49.1	747 ± 22.7	785 ± 72.4	780 ± 29.8	850 ± 47.8
S0.25	900 ± 21.7	830 ± 22.9	772 ± 29.7	797 ± 32.1	794 ± 15.6	900 ± 21.7
S0.5	912 ± 14.1	816 ± 24.1	775 ± 50.8	802 ± 37.6	783 ± 32.3	912 ± 14.1
S1.0	874 ± 31.0	814 ± 79.2	755 ± 84.8	764 ± 65.4	762 ± 34.1	874 ± 31.0

Statistical evaluation revealed no significant differences between the groups (*p* > 0.05).

1 CON, control (basal diet without microalgae); C0.5, C1.0, C2.0, basal diet supplemented with *Chlorella vulgaris* at 0.5%, 1.0%, and 2.0%, respectively; S0.25, S0.5, S1.0, basal diet supplemented with *Schizochytrium limacinum* at 0.25%, 0.5%, and 1.0%, respectively.

### Foot pad health and animal losses

Foot pad dermatitis scores remained very low throughout the study (Table 8). All scores indicated minimal to no lesions, reflecting excellent litter conditions and foot health. Statistical analysis confirmed no significant differences between dietary treatments at any sampling point (*p* > 0.05). No animal losses or health impairments requiring veterinary intervention occurred during the experimental period.

**Table 8:** Foot pad scoring during the experimental period (Mean ± SD).

Group^1^	Day 7	Day14	Day 21*	Day 28*	Day 35*
CON	0.13 ± 0.27	0.48 ± 0.39	1.00	1.00	1.00
C0.5	0.25 ± 0.34	0.69 ± 0.37	1.00	1.00	1.00
C1.0	0.16 ± 0.26	0.83 ± 0.24	1.00	1.00	1.00
C2.0	0.14 ± 0.25	0.70 ± 0.30	1.00	1.00	1.00
S0.25	0.15 ± 0.30	0.71 ±0.30	1.00	1.00	1.00
S0.5	0.19 ± 0.37	0.55 ± 0.37	1.00	1.00	1.00
S1.0	0.24 ± 0.38	0.64 ± 0.34	1.03 ± 0.11	1.00	1.00

Statistical evaluation revealed no significant differences between the groups (*p* > 0.05).

⁎ SD = 0 indicates all observations within this group were identical.

1 CON, control (basal diet without microalgae); C0.5, C1.0, C2.0, basal diet supplemented with *Chlorella vulgaris* at 0.5%, 1.0%, and 2.0%, respectively; S0.25, S0.5, S1.0, basal diet supplemented with *Schizochytrium limacinum* at 0.25%, 0.5%, and 1.0%, respectively.

### Serum immunology

To assess the success of routine vaccination, the serum antibody response to NDV was evaluated at the end of the trial (day 36) (Table 9). The proportion of broilers exhibiting positive anti-NDV antibody titers was significantly influenced by dietary microalgae supplementation (*p* < 0.05). Specifically, birds fed the diet containing 0.5% *Schizochytrium limacinum* (S0.5) showed a significantly higher seropositivity rate (100%) compared to the CON (66.7%). Furthermore, the seropositivity rate in the S0.5 group was also statistically greater than that observed in the group receiving 2.0% *Chlorella vulgaris* (C2.0). These results suggest that dietary inclusion of *Schizochytrium limacinum* at 0.5% may enhance the humoral immune response to NDV vaccination in broilers under the conditions of this study.

**Table 9:** Seropositivity to Newcastle Disease Virus (NDV) antibodies in broiler serum at day 36, depending on dietary treatment.

Group	Positive, n (%)	Negative, n (%)
CON	10 (66.7)	5 (33.3)
C0.5	12 (80.0)	3 (20.0)
C1.0	12 (80.0)	3 (20.0)
C2.0	9 (60.0)	6 (40.0)
S0.25	11 (73.3)	4 (26.7)
S0.5	15 (100)	0 (0.0)
S1.0	11 (73.3)	4 (26.7)

*n* = 15 per group. CON, control; C0.5–C2.0, *Chlorella vulgaris* at 0.5–2.0%; S0.25–S1.0, *Schizochytrium limacinum* at 0.25–1.0%. Group comparisons were performed using Fisher's exact test (pairwise); the proportion of seropositive birds in S0.5 was significantly higher than in CON (*p* = 0.0421) and C2.0 (*p* = 0.0169). No other pairwise comparisons were significant.

### Intestinal morphology

Histomorphometric parameters of the jejunum and ileum are presented in Table 10. No significant differences were observed between any of the dietary treatment groups and the CON for VH, VW, CD, or the VH:CD ratio in either intestinal segment (*p* > 0.05).

**Table 10:** Jejunum and ileum histopathological parameters of broilers at 36 days of age depending on the feeding (given in µm, Mean ± SD).

Group^1^	Villus height	Villus width	Crypt depth	Villus length to crypt depth ratio
Jejunum				
CON	1219 ± 148	110 ± 45.4	139 ± 42.5	9.28 ± 2.11
C0.5	1221 ± 153	115 ± 39.1	128 ± 29.0	9.85 ± 1.74
C1.0	1188 ± 110	117 ± 49.0	134 ± 19.9	9.01 ± 1.31
C2.0	1160 ± 274	140 ± 68.9	121 ± 26.5	9.86 ± 2.78
S0.25	1231 ± 135	94.9 ± 32.3	142 ± 24.4	8.98 ± 2.24
S0.5	1203 ± 196	115 ± 52.7	139 ± 28.5	8.93 ± 2.14
S1.0	1220 ± 107	113 ± 48.4	144 ± 31.0	8.86 ± 2.23
Ileum				
CON	735 ± 106	107 ± 24.8	99.1 ± 19.4	7.63 ± 1.66
C0.5	778 ± 109	105 ± 24.0	99.6 ± 14.5	8.05 ± 1.96
C1.0	766 ± 87.7	110 ± 27.5	93.1 ± 17.1	8.47 ± 1.83
C2.0	778 ± 134	107 ± 24.2	99.3 ± 14.9	8.00 ± 1.84
S0.25	768 ± 83.4	108 ± 29.6	103 ± 21.1	7.63 ± 1.29
S0.5	821 ± 154	95.9 ± 17.9	94.0 ± 18.4	8.93 ± 1.75
S1.0	796 ± 97.7	122 ± 28.7	102 ± 19.0	7.98 ± 1.51

Statistical evaluation revealed no significant differences between the groups (*p* > 0.05).

1 CON, control (basal diet without microalgae); C0.5, C1.0, C2.0, basal diet supplemented with *Chlorella vulgaris* at 0.5%, 1.0%, and 2.0%, respectively; S0.25, S0.5, S1.0, basal diet supplemented with *Schizochytrium limacinum* at 0.25%, 0.5%, and 1.0%, respectively.

### Serum and hepatic fatty acids profile

The serum fatty acid composition at day 36 was markedly affected by *Schizochytrium limacinum* supplementation, particularly with respect to several long-chain polyunsaturated fatty acids (Table 11). The most pronounced effects were observed in the levels of long-chain PUFAs. Serum docosahexaenoic acid (DHA, C22:6 n-3) concentration increased markedly from 18.3 μg/mL in the CON group to 161 μg/mL in the S1.0 group, representing an approximately nine-fold increase (p < 0.05). Eicosapentaenoic acid (EPA, C20:5 n-3) also increased significantly with increasing *Schizochytrium limacinum* inclusion. Conversely, serum arachidonic acid (C20:4 n-6) concentrations were significantly lower in all *Schizochytrium limacinum*-supplemented groups compared with the CON group (p < 0.05). Supplementation with *Chlorella vulgaris* did not significantly alter the concentrations of these key PUFAs in serum compared with the CON diet.

**Table 11:** Levels of different fatty acids contents (µg/mL) in serum of birds at end of trial (Mean ± SD).

Group^1^	Palmitic acid	Stearic acid	γ-Linolenic acid	α-Linolenic acid	Eicosadienoic acid	Arachidonic acid	Eicosapentae-noic acid	Docosahexaenoic acid
CON	567^a^ ± 76.2	413^a^^,^^b^^,^^c^ ± 60.9	4.62^a^ ± 1.20	21.7^b^^,^^c^ ± 3.41	12.4^a^ ± 1.64	206^a^ ± 32.9	9.07^c^ ± 2.28	18.3^d^ ± 3.60
C0.5	602^a^ ± 59.0	458^a^ ± 58.5	4.46^a^ ± 1.31	24.5^a^^,^^b^ ± 3.52	13.2^a^ ± 2.22	209^a^ ± 37.9	11.8^b^^,^^c^ ± 2.72	34.9^d^ ± 28.1
C1.0	559^a^ ± 52.4	440^a^^,^^b^ ± 41.4	4.44^a^ ± 1.01	21.7^b^^,^^c^ ± 2.84	13.0^a^ ± 1.56	223^a^ ± 40.9	9.18^c^ ± 2.15	24.0^d^ ± 6.51
C2.0	566^a^ ± 82.3	437^a^^,^^b^ ± 75.0	4.50^a^ ± 1.03	25.6^a^ ± 3.32	12.5^a^ ± 2.25	203^a^ ± 41.3	10.7^c^ ± 2.50	21.0^d^ ± 5.53
S0.25	581^a^ ± 75.6	398^b^^,^^c^ ± 52.2	3.89^a^^,^^b^ ± 0.84	21.0^b^^,^^c^ ± 4.05	11.4^a^^,^^b^ ± 1.60	159^b^ ± 27.8	11.9^b^^,^^c^ ± 2.43	81.7^c^ ± 13.6
S0.5	564^a^ ± 52.4	366^c^ ± 30.0	3.18^b^^,^^c^ ± 0.75	20.8^b^^,^^c^ ± 3.52	10.0^b^ ± 1.78	138^b^ ± 28.3	15.1^b^ ± 5.02	117^b^ ± 21.0
S1.0	598^a^ ± 64.7	384^c^ ± 63.7	2.77^c^ ± 1.27	20.4^c^ ± 4.48	10.2^b^ ± 1.35	138^b^ ± 50.5	20.4^a^ ± 7.05	161^a^ ± 43.4

a Different superscripts within column mark significant differences between the groups (*p* < 0.05).

b Different superscripts within column mark significant differences between the groups (*p* < 0.05).

c Different superscripts within column mark significant differences between the groups (*p* < 0.05).

d Different superscripts within column mark significant differences between the groups (*p* < 0.05).

1 CON, control (basal diet without microalgae); C0.5, C1.0, C2.0, basal diet supplemented with *Chlorella vulgaris* at 0.5%, 1.0%, and 2.0%, respectively; S0.25, S0.5, S1.0, basal diet supplemented with *Schizochytrium limacinum* at 0.25%, 0.5%, and 1.0%, respectively.

The fatty acid profile in liver tissue (Table 12) mirrored the trends observed in serum, demonstrating parallel dose-dependent responses to *Schizochytrium* supplementation. Hepatic DHA content increased steeply with dietary SI inclusion, reaching 6.21 g/kg DM in the S1.0 group, compared to 0.80 g/kg in the CON (*p* < 0.05). A concurrent, significant reduction in hepatic arachidonic acid content was observed in the S0.5 and S1.0 groups compared to the CON. Supplementation with *Chlorella vulgaris* had minimal impact on the hepatic levels of these PUFAs.

**Table 12:** Levels of different fatty acids contents (g/kg dry matter) in liver of birds at end of trial (Mean ± SD).

Group^1^	Palmitic acid	Stearic acid	γ-Linolenic acid	α-Linolenic acid	Eicosadienoic acid	Arachidonic acid	Eicosapentae-noic acid	Docosahexaenoic acid
CON	25.4^a^ ± 8.59	19.6^b^ ± 3.48	0.12^a^ ± 0.04	0.50^b^ ± 0.11	0.61^b^ ± 0.07	8.44^a^ ± 1.20	0.22^a^ ± 0.03	0.80^d^ ± 0.19
C0.5	30.3^a^ ± 13.6	20.8^b^ ± 4.23	0.12^a^ ± 0.04	0.52^b^ ± 0.13	0.62^b^ ± 0.16	7.72^a^^,^^b^ ± 0.92	0.25^a^ ± 0.05	1.05^d^ ± 0.28
C1.0	27.9^a^ ± 13.4	21.2^b^ ± 4.56	0.12^a^ ± 0.02	0.48^b^ ± 0.11	0.64^b^ ± 0.10	8.72^a^ ± 1.35	0.22^a^ ± 0.06	1.0^d^ ± 0.22
C2.0	28.9^a^ ± 12.5	21.2^b^ ± 3.80	0.12^a^ ± 0.02	0.57^b^ ± 0.11	0.62^b^ ± 0.08	8.19^a^^,^^b^ ± 1.65	0.27^a^ ± 0.05	0.94^d^ ± 0.28
S0.25	39.3^a^ ± 23.6	23.7^b^ ± 7.77	0.11^a^ ± 0.03	0.53^b^ ± 0.15	0.64^b^ ± 0.10	6.95^b^^,^^c^ ± 1.44	0.27^a^ ± 0.04	3.41^c^ ± 0.63
S0.5	40.3^a^ ± 23.7	24.8^a^^,^^b^ ± 9.75	0.10^a^ ± 0.03	0.56^b^ ± 0.23	0.65^b^ ± 0.15	6.10^c^^d^ ± 1.27	0.34^b^ ± 0.11	4.96^b^ ± 0.84
S1.0	59.0^a^ ± 34.3	31.4^a^ ± 12.5	0.12^a^ ± 0.04	0.78^a^ ± 0.33	0.79^a^ ± 0.22	5.11^d^ ± 1.24	0.39^b^ ±0.13	6.21^a^ ± 1.18

a Different superscripts within column mark significant differences between the groups (*p* < 0.05).

b Different superscripts within column mark significant differences between the groups (*p* < 0.05).

c Different superscripts within column mark significant differences between the groups (*p* < 0.05).

d Different superscripts within column mark significant differences between the groups (*p* < 0.05).

1 CON, control (basal diet without microalgae); C0.5, C1.0, C2.0, basal diet supplemented with *Chlorella vulgaris* at 0.5%, 1.0%, and 2.0%, respectively; S0.25, S0.5, S1.0, basal diet supplemented with *Schizochytrium limacinum* at 0.25%, 0.5%, and 1.0%, respectively.

### Meat quality parameters

Instrumental color and quality parameters of breast meat (*Musculus pectoralis superficialis*) are shown in Table 13. The yellowness (b) value was significantly higher in fillets from the C2.0 group (17.0) compared to the CON (6.50) (*p* < 0.05). This intensified yellow coloration was also visually apparent. Electrical conductivity (EC) was significantly higher in breast meat from the C2.0 group (7.55) compared to the CON (5.00) (*p* < 0.05). No significant differences were found among treatments for meat pH, lightness (L value), or redness (a* value).

**Table 13:** Breast meat quality parameters of birds at end of trial (Mean ± SD).

Group^1^	pH value	Electrical conductivity	L* (Lightness)	a* (Redness)	b* (Yellowness)
CON	5.56^a^ ± 0.05	5.00^b^ ± 0.20	62.5^a^ ± 0.30	3.50^a^ ± 0.10	6.50^b^ ± 0.50
C0.5	5.75^a^ ± 0.04	6.65^a^^,^^b^ ± 0.60	62.0^a^ ± 0.30	3.70^a^ ± 0.30	11.3^b^ ± 1.0
C2.0	5.73^a^ ± 0.05	7.55^a^ ± 0.55	62.0^a^ ± 0.30	1.80^a^ ± 0.90	17.0^a^ ± 0.80
S0.25	5.76^a^ ± 0.04	6.15^a^^,^^b^ ± 0.55	61.0^a^ ± 0.30	2.10^a^ ± 1.00	7.50^b^ ± 0.30
S1.0	5.74^a^ ± 0.05	6.65^a^^,^^b^ ± 0.15	60.3^a^ ± 0.50	3.30^a^ ± 0.30	8.50^b^ ± 1.80

a Different superscripts within column mark significant differences between the groups (*p* < 0.05).

b Different superscripts within column mark significant differences between the groups (*p* < 0.05).

1 CON, control (basal diet without microalgae); C0.5, C1.0, C2.0, basal diet supplemented with *Chlorella vulgaris* at 0.5%, 1.0%, and 2.0%, respectively; S0.25, S0.5, S1.0, basal diet supplemented with *Schizochytrium limacinum* at 0.25%, 0.5%, and 1.0%, respectively.

## Discussion

This study investigated the dose-dependent effects of two microalgae species, *Chlorella vulgaris* and *Schizochytrium limacinum*, on broiler production parameters. The algae exerted distinct, species-specific effects that were strongly influenced by inclusion level, extending our prior screening study, which identified species-specific impacts on meat color and fatty acid profiles at a fixed 1% inclusion rate (Abd El-Wahab et al., 2025).

### Feed intake and growth performance

The 0.5% inclusion of Chlorella vulgaris (C0.5) significantly improved final BW and ADG relative to CON, a 10% improvement over Ross 308 performance goals Aviagen (2022), achieved without compromising feed conversion. This contrasts with our previous study, where no growth differences were found among algae-fed groups at 1% (Abd El-Wahab et al., 2025), suggesting lower Chlorella levels may better provide optimal bioactive compounds (Kang et al., 2013).

The mechanism underlying this growth promotion remains unclear. Chlorella vulgaris contains bioactive compounds such as β-glucans, associated with immunomodulatory and gut health effects in poultry (Kang et al., 2017; Lee et al., 2023). The absence of a similar benefit at higher inclusion levels implies a plateau or negative threshold effect: increased non-starch polysaccharide content may elevate digesta viscosity (8.30 mPas vs. 2.0 mPas in diets with 10% vs. 0% rye), reducing nutrient absorption efficiency (Bedford and Classen, 1992; Van Krimpen et al., 2017), while excessive β-glucan stimulation may divert metabolic energy from growth toward immune function (Spínola et al., 2024). This is consistent with reports that high inclusion of microalgae with intact cell walls or high non-starch polysaccharide content can reduce nutrient digestibility or shift energy partitioning away from growth (Pestana et al., 2020).

*Schizochytrium limacinum* did not enhance growth performance, and the S0.5 group showed a transient BW reduction during the grower phase. This may reflect an adaptation period to a potentially altered dietary fatty acid profile; however, as the omega-3 content of the microalgae and final diets was not directly analyzed, this remains a hypothesis rather than a confirmed mechanism. No comparable transient effect occurred with *Chlorella vulgaris*, which lacks high omega-3 lipid content, lending indirect support to this interpretation. Similar short-term adjustments following abrupt dietary lipid changes have been reported elsewhere (Baéza et al., 2015), and this aligns with our earlier finding that 1% *Schizochytrium limacinum* did not affect final BW (Abd El-Wahab et al., 2025). The long-chain PUFAs in *Schizochytrium*, particularly DHA, may alter nutrient partitioning rather than directly promoting growth (Long et al., 2018). The absence of FCR differences across treatments, consistent with previous reports on moderate microalgae inclusion (Pestana et al., 2020), indicates that the benefits observed (growth in C0.5, fatty acid enrichment in *Schizochytrium* groups) did not come at the cost of feed efficiency.

### Excreta/litter dry matter, foot pad health, and animal welfare

Foot pad health and litter quality remained excellent and unaffected across all groups, with dermatitis scores indicating minimal to no lesions throughout the study (Mayne et al., 2007). The absence of differences in excreta and litter DM content indicates that neither microalgae substantially altered digestive water balance or intestinal transit. No animal losses or health problems requiring intervention occurred, supporting the safety and palatability of both microalgae at the tested levels.

### Serum immunology

The enhanced seroconversion rate in the S0.5 group (100% vs. 66.7% in CON) likely reflects omega-3 PUFA-mediated modulation of B-lymphocyte function and antibody production (Al-Khalaifah and Al-Nasser, 2021), consistent with the anti-inflammatory shift in fatty acid profile associated with Schizochytrium limacinum supplementation. That this effect occurred at the intermediate (0.5%) but not the highest (1.0%) inclusion level suggests a non-linear relationship, possibly reflecting an optimal shift in the omega-6/omega-3 ratio for immune cell function (Calder, 2017; Sun et al., 2018), warranting further investigation across a broader range of immune parameters.

### Intestinal morphology

Consistent with our earlier findings (Abd El-Wahab et al., 2025), neither algal species at any tested dose significantly altered jejunal or ileal morphology (VH, VW, CD, and VH:CD ratio), suggesting these algae do not impair nutrient absorption capacity within the tested range under optimal rearing conditions. This may partly reflect the excellent hygiene and low pathogen challenge of the trial environment, under which intestinal architecture is typically already well-developed, potentially limiting observable treatment benefit - in contrast to reports of improved morphology following microalgae supplementation under different conditions (Mirzaie et al., 2020; Chang et al., 2021).

Histomorphology represents only one dimension of intestinal health; effects on gut microbiota, local immune populations, and barrier function remain uncharacterized (Lee et al., 2023; Liu et al., 2021) and should be addressed in future studies incorporating microbiome analysis and functional barrier markers.

### Systemic and hepatic fatty acid profiles

The most pronounced effects of this study were in serum and liver fatty acid composition. *Schizochytrium limacinum* produced a linear increase in DHA and concurrent decrease in arachidonic acid, confirming and quantifying the trend from our previous screening (serum DHA of 117 μg/mL at 1% vs. 16.7–18.9 μg/mL in controls;(Abd El-Wahab et al., 2025). The present dose-response data (serum DHA rising nearly nine-fold, liver DHA over seven-fold, from CON to 1% inclusion) demonstrate the exceptional efficiency of *Schizochytrium* in enriching broiler tissues with omega-3 PUFAs, consistent with Long et al. (2018), and support targeted enrichment of broiler products to meet specific market demands.

The concurrent reduction in arachidonic acid, a precursor to pro-inflammatory eicosanoids (Pereira et al., 2024; Sala et al., 2018), alongside the increase in anti-inflammatory omega-3 PUFAs like DHA and EPA, suggests a potential shift in the birds' inflammatory tone toward a more anti-inflammatory state (Calder, 2017), with potential relevance to bird health under stress conditions not present in this optimal-condition trial.

This efficient feed-to-tissue omega-3 transfer represents a sustainable strategy for enhancing the nutritional value of poultry meat, given the cardiovascular and anti-inflammatory benefits associated with dietary DHA (Banaszak et al., 2024; Calder, 2017; Sun et al., 2018). Notably, even the lowest inclusion level produced a marked increase in hepatic DHA, indicating high bioavailability of *Schizochytrium*-derived DHA, consistent with prior reports in poultry.

In contrast, *Chlorella vulgaris*, even at 2%, had minimal effects on serum and hepatic DHA, EPA, or arachidonic acid - consistent with its comparatively low long-chain PUFA content. *Chlorella's* functional properties in animal nutrition are instead generally attributed to pigments, proteins, and polysaccharides (Ritu et al., 2023), reflecting the fundamental compositional difference between the two microalgae.

### Meat quality: pigmentation vs. fatty acid enrichment

*Chlorella vulgaris* significantly increased breast meat yellowness (b*) in a dose-dependent manner, most pronounced in C2.0 (17.0 vs. 6.50 in CON), extending our previous finding of elevated b* with opened-cell-wall *Chlorella* at 1% (Abd El-Wahab et al., 2025). This is attributed to deposition of algal carotenoids such as lutein and β-carotene - present in substantial quantities in *Chlorella vulgaris* alongside chlorophyll (Alfaia et al., 2021; Pestana et al., 2020) which animals cannot synthesize de novo (Maoka, 2020), so their presence in muscle directly reflects dietary intake. Therefore, dietary supplementation with *Chlorella vulgaris* may offer a natural strategy for enhancing breast-meat yellowness, which can influence consumer acceptance, although preferences for meat color vary considerably across markets and cultures (Altmann et al., 2023), with carotenoids additionally serving as vitamin precursors with antioxidant properties (Alfaia et al., 2021; Christaki et al., 2015; Ritu et al., 2023).

The elevated EC in C2.0 breast meat (7.55 vs. 5.00 in CON) - occurring without a corresponding pH change, unlike the typical inverse relationship between these parameters (Joo et al., 2013; Lee et al., 2022) - reproduces a finding from our initial screening study (Abd El-Wahab et al., 2025), and warrants further investigation, potentially including water-holding capacity and drip loss measurements.

*Schizochytrium limacinum*, while drastically altering tissue fatty acid profiles, had no significant effect on breast meat color. This finding was expected, given that *Schizochytrium* is cultivated heterotrophically without light and therefore contains minimal chlorophyll or carotenoid pigments (Valente et al., 2021). The enrichment of meat with DHA through *Schizochytrium* supplementation represents a powerful tool for creating functional poultry products with enhanced nutritional value for human consumers, offering potential cardiovascular and anti-inflammatory benefits (Banaszak et al., 2024; Calder, 2017).

### Practical implications and optimal inclusion levels

The results of this dose-response study provide practical guidance for commercial application. For *Chlorella vulgaris*, 0.5% inclusion appears optimal for growth promotion, balancing performance benefits against the cost of higher inclusion. At 2.0%, while meat pigmentation is maximized, this comes at higher feed cost without additional growth benefit and with potential changes in meat quality (elevated EC) requiring further evaluation.

For *Schizochytrium limacinum*, the optimal level depends on the production goal: 0.25% may suffice for modest omega-3 enrichment with minimal disruption to other parameters, while 0.5-1.0% is more appropriate for substantial DHA enrichment targeting a differentiated "omega-3 enriched" product. Although liver DHA deposition responded strongly even at 0.25%, achieving the higher tissue concentrations needed for a marketable product likely requires these higher levels.

The choice between these algae must balance biological efficacy with economic viability. *Schizochytrium limacinum* is a premium ingredient due to its specialized cultivation requirements, but its substantial DHA enrichment even at 0.25-0.5% may justify the cost for producers targeting premium, health-enhanced poultry markets. Conversely, the growth-promoting effect of low-dose *Chlorella vulgaris* (0.5%) could offset ingredient costs through improved feed efficiency and shortened production cycles. Economic analysis weighing these costs against the premium value of omega-3 enriched products would be needed to determine commercial viability.

### Study limitations and future perspectives

The trial was conducted under optimal environmental conditions with minimal stressors, which may have limited the expression of health-protective effects; under commercial conditions with heat, pathogen, or nutritional stress, the immunomodulatory and antioxidant effects of these microalgae might be more apparent. Direct analysis of breast meat fatty acid composition was not performed; given the substantial tissue enrichment observed in liver and serum, corresponding meat omega-3 analysis would strengthen the translational relevance of these findings for human nutrition. Carcass yield parameters (e.g., dressing percentage, breast meat yield) and consumer sensory evaluation were also not assessed, despite birds being available at slaughter, and are recommended as priorities for future studies alongside economic and consumer-acceptance analysis of pigment-enhanced and omega-3 enriched products. The absence of microbiome analysis, digestibility measurements, or molecular markers of immune function further limits our ability to fully explain the mechanisms underlying the observed effects.

Additionally, because both microalgae were included on top of an identical basal diet without compensatory reformulation, the experimental diets were not confirmed to be strictly isocaloric or isonitrogenous across treatments; since the analyzed diet composition (Table 1) reflects only the basal diet, the nutrient contribution of the algae themselves could not be precisely quantified. Future studies should formulate iso-energetic, iso-nitrogenous diets across all inclusion levels to more clearly separate the algae's functional bioactivity from their inherent nutrient contribution.

## Conclusions

This study demonstrates that *Chlorella vulgaris* and *Schizochytrium limacinum* exert distinct, dose-dependent effects on broiler performance, immune response, and product quality, reflecting their fundamentally different biochemical compositions. Low-dose *Chlorella vulgaris* supplementation favors growth performance, whereas higher inclusion levels shift its functional role toward meat pigmentation. *Schizochytrium limacinum*, by contrast, acts primarily as an efficient omega-3 delivery vehicle, with additional potential to support immune function.

These findings indicate that the optimal application of each microalga depends on the intended production goal - growth promotion, meat pigmentation, or omega-3 enrichment - rather than a single universally optimal inclusion level. This targeted-use framework offers commercial broiler producers a practical basis for developing differentiated, functionally enhanced poultry products through strategic microalgae supplementation.

Future research should focus on cost-benefit analyses of these applications, investigate the effects of combining both algae to achieve multiple benefits simultaneously, validate these findings under commercial-scale rearing conditions, and explore the underlying mechanisms of the observed dose-dependent responses through microbiome, metabolomic, and molecular analyses. For practical application, 0.5% *Chlorella vulgaris* is recommended for growth promotion, 2.0% for pigmentation, and 0.5-1.0% *Schizochytrium limacinum* for omega-3 enrichment of tissues.

## CRediT authorship contribution statement

**Jan Berend Lingens:** Writing – review & editing, Writing – original draft, Visualization, Methodology, Investigation, Formal analysis. **Dana-Carina Schubert:** Writing – review & editing, Project administration, Investigation, Conceptualization. **Svenja Starke:** Writing – review & editing, Funding acquisition, Conceptualization. **Carsten Krischek:** Writing – review & editing, Methodology. **Christian Visscher:** Writing – review & editing, Writing – original draft, Supervision, Methodology, Funding acquisition, Conceptualization. **Amr Abd El-Wahab:** Writing – review & editing, Writing – original draft, Visualization, Supervision, Methodology, Investigation.

## Disclosures

The authors declare that they have no conflicts of interest and there has been no significant financial support for this work that could have influenced its outcome. The project was sponsored by the German Federal Environmental Foundation. (Gefördert durch die Deutsche Deutsche Bundesstiftung Umwelt, DBU)..

We acknowledge financial support by the Open Access Publication Fund of the University of Veterinary Medicine Hannover, Foundation.
